# Supplementary material for: Endurance Training in Humans Modulates the Bacterial DNA Signature of Skeletal Muscle
Source: Biomedicines. 2021 Dec 29;10(1):64. doi: 10.3390/biomedicines10010064 (PMC8773292; doi:10.3390/biomedicines10010064)
Supplement: Supplementary file 1 [file biomedicines-10-00064-s001.zip › biomedicines-1436413-supplementary.pdf]

SUPPLEMENTAL MATERIAL

## Supplementary Tables

**Table S1: 16S copy numbers per ng of extracted DNA across samples.** We present the data ±SD.

| | **16S copy numbers per ng DNA** |
|---|---|
| **Muscle Untrained (N=8)** | 1258 ± 179 |
| **Muscle Trained (N=8)** | 1257 ± 89 |
| **Blood Untrained (N=6)** | 852 ± 123 |
| **Blood Trained (N=6)** | 983 ± 164 |
| **Muscle-EXT-NC (N=2)** | 97 ± 8 |
| **Blood-EXT-NC (N=4)** | 75 ± 25 |
| **qPCR-NC (N=1)** | 35 |

**Table S2**: According to the gain in $VO_2$ max after training, the training efficiency of participants was classified as *High* (ΔVO2max > 12), *Medium* (6 < ΔVO2max <12) or *Low* (ΔVO2max < 6).

| **Participant ID** | **$\Delta VO_2$ max (pre-post training)** | **Training Efficiency** |
|---|---|---|
| 11 | 9,94 | Medium |
| 15 | 12,84 | High |
| 16 | 15,85 | High |
| 17 | 10,65 | Medium |
| 18 | 12,93 | High |
| 20 | 1,95 | Low |
| 24 | 8,25 | Medium |
| 25 | 5,29 | Low |

## Supplementary Figures and Figure Legends

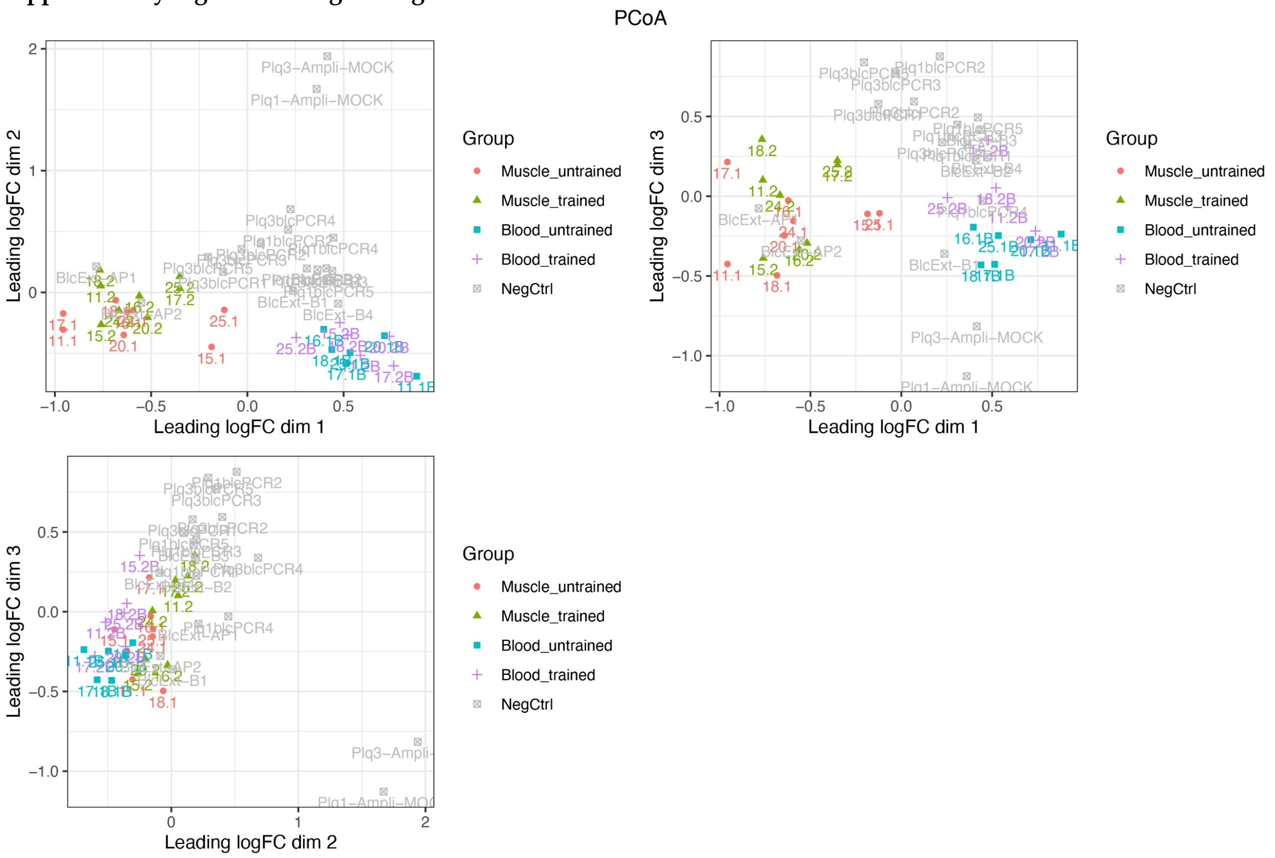


**Figure S1:** Multidimensional Analysis plot of samples and negative controls.

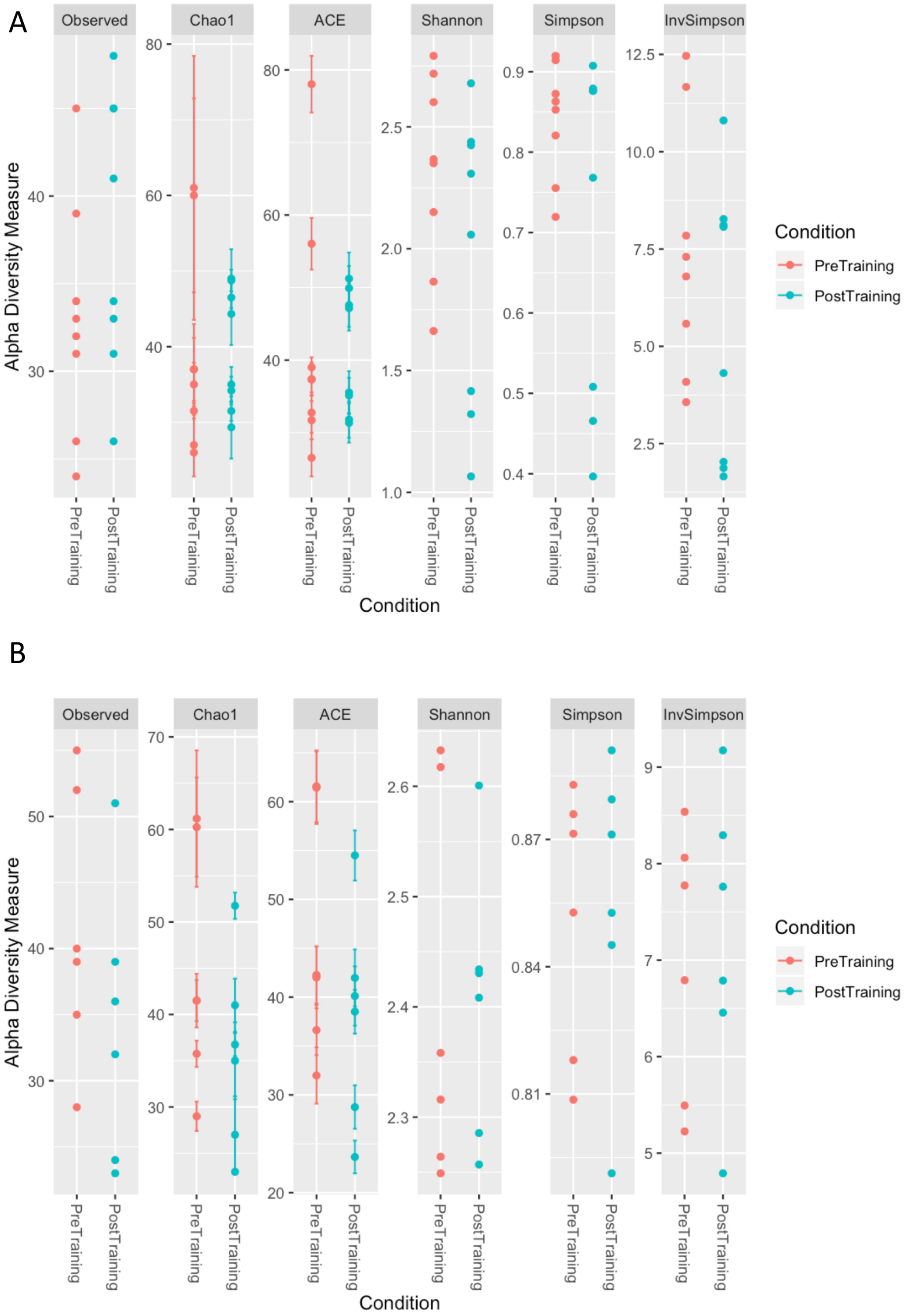


**Figure S2:** Alpha diversities in muscle (A) and blood (B).

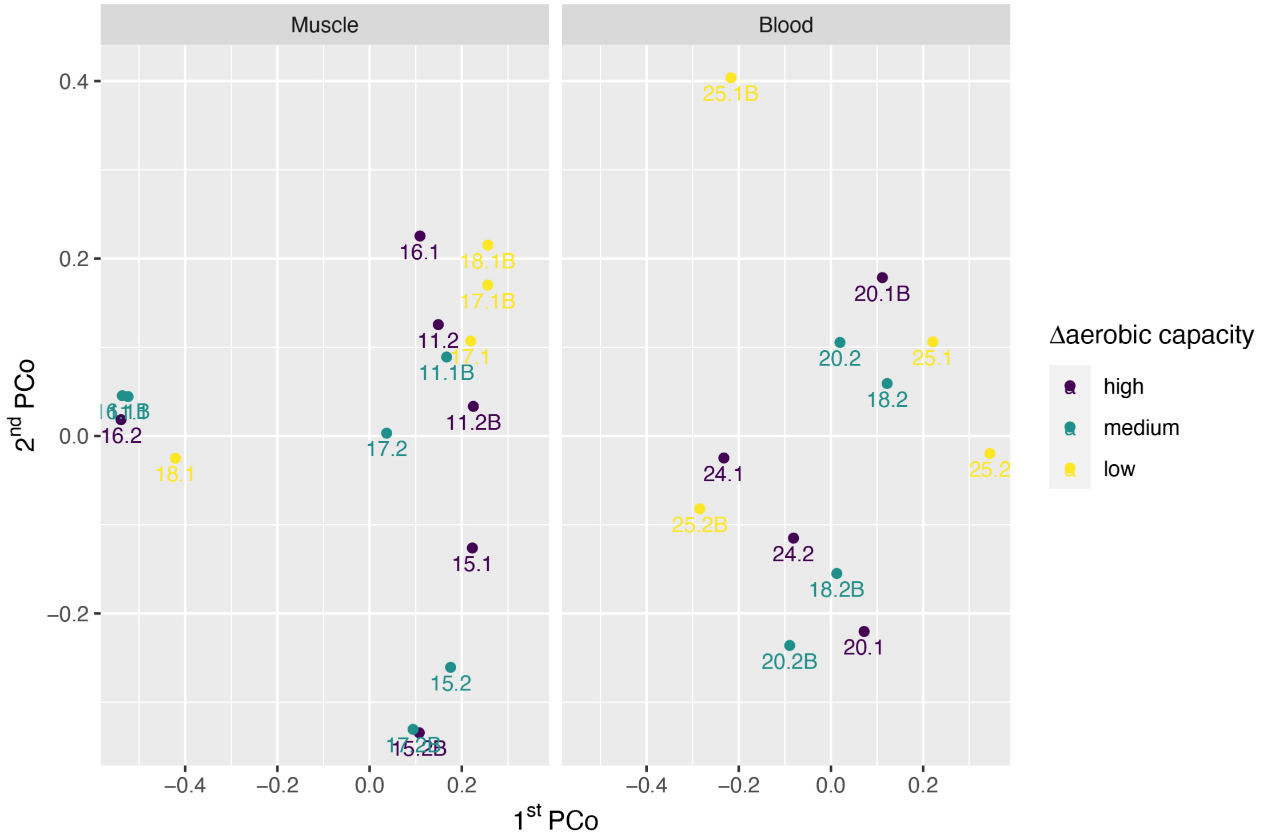


**Figure S3:** Multidimensional Analysis plot of samples coloured according to the aerobic capacity differences between untrained and trained conditions.
